# Sonographic detection of pneumoperitoneum

**DOI:** 10.1259/bjrcr.20160146

**Published:** 2017-05-04

**Authors:** Marlom Khor, Joshua Cutten, Joel Lim, Yuranga Weerakkody

**Affiliations:** ^1^General Surgery, Fiona Stanley Hospital, Murdoch, Perth, WA, Australia; ^2^Radiology, Royal Perth Hospital, Perth, WA, Australia

## Abstract

Accurate and timely detection of a perforated hollow viscus is crucial and has profound consequences for the patient with an acute abdomen. While a CT scan can provide an accurate diagnosis, the increasingly indiscriminate use of this modality sparks concern regarding radiation dosing, its associated safety concerns and its timely occurrence. There are distinct and readily reproducible findings of pneumoperitoneum on ultrasound. However, sonographers should be trained to detect pneumoperitoneum or patients may be discharged with false-negative results. This case report supports such a view and investigates the current literature surrounding this issue.

## Case report—structure

An intra-abdominal hollow viscus perforation can be fatal if left undiagnosed and untreated. Ultrasound findings, while sensitive, are rarely utilized in current acute clinical settings. We present a case of a 29-year-old male who was admitted to the emergency department with right upper quadrant abdominal pain. An abdominal ultrasound was able to identify distinct echogenic stripe-like bands overlying the splenic and hepatic convexities (findings indicative of a pneumoperitoneum). This was subsequently confirmed on a chest radiograph.

## Clinical presentation

A 29-year-old male presented to the emergency department with epigastric pain radiating to his right iliac fossa. He had described the pain as being most pronounced in the right upper abdominal region which, on initial history taking and examination, suggested a colicky nature. This was on a background of heavy alcohol intake a week prior to presentation. He denied anti-inflammatory use or other systemic symptoms. The patient had no previous relevant imaging or a history of any pre-existing medical condition.

## Imaging findings

**Figure 1.**
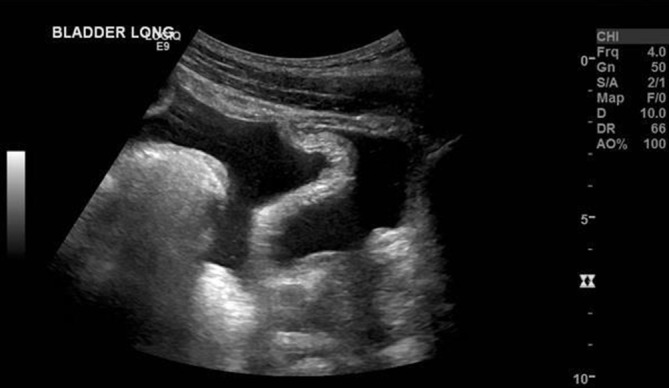
A midsagittal image documenting a partially distended urinary bladder, with dependant-free fluid in the pelvis.

**Figure 2.**
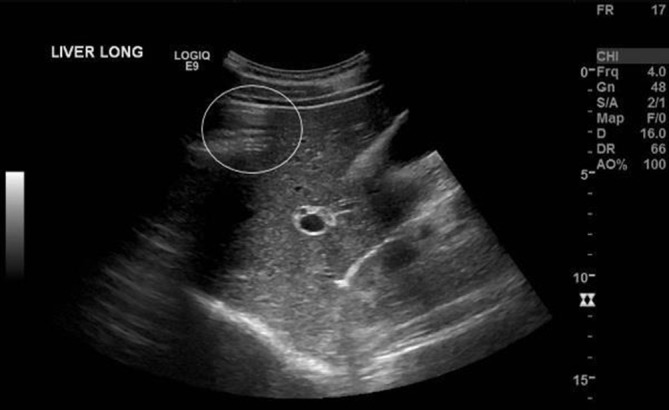
Sagittal image of the liver through segments 8 and 5. The peritoneal stripe sign can be appreciated obscuring the superior aspect of segment 8. There was no appearance of gallstones and Murphy’s sign was negative on ultrasound probe pressure.

**Figure 3.**
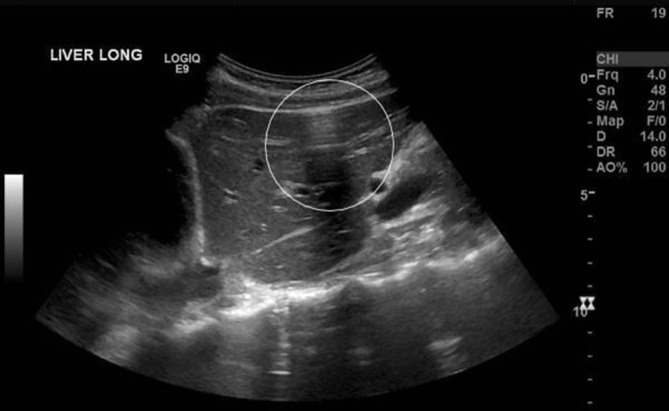
A sagittal image documented through segments 4a, 4b and 1. The peritoneal stripe sign has been indicated. This image is the most convincing because it is taken in the midline—clear of ribs, bowel and lung artefactual distortions.

**Figure 4.**
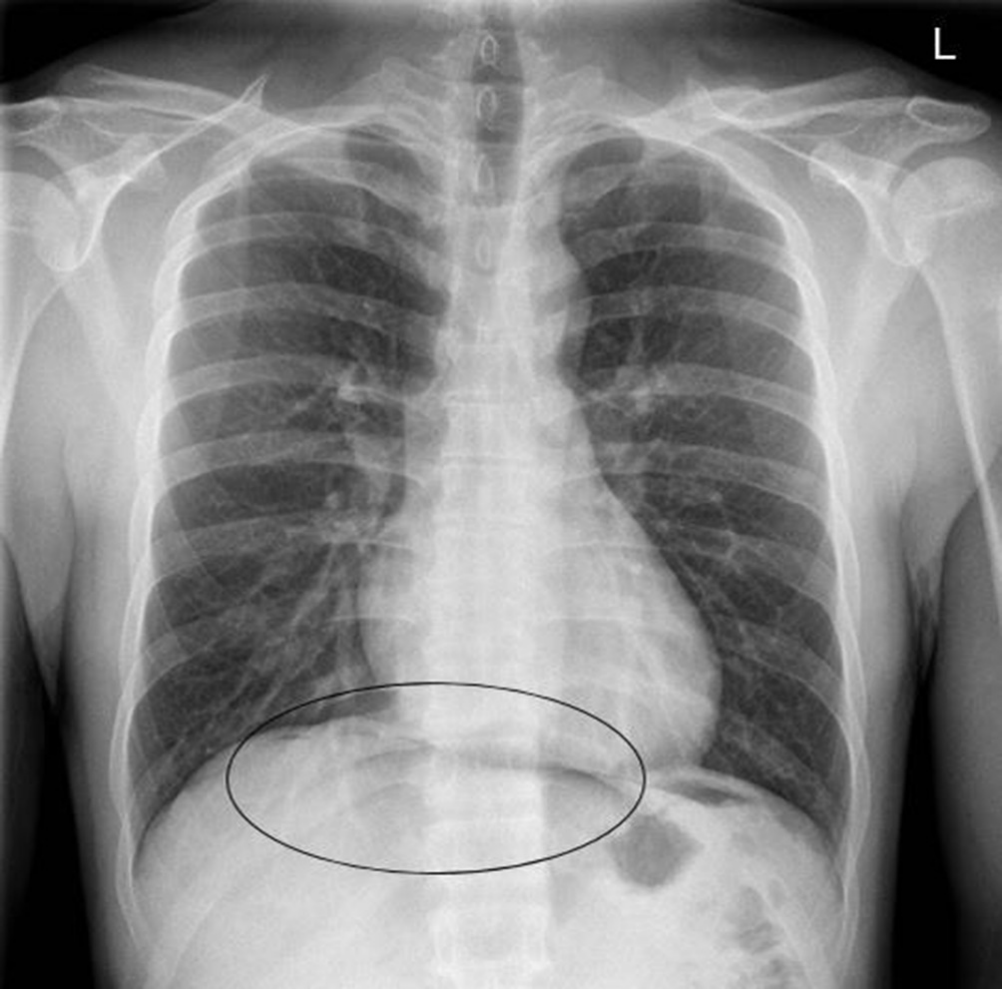
A PA erect chest x-ray; confirming the ultrasound findings and the presence of subdiaphragmatic free air (pneumoperitoneum).

An abdominal ultrasound was initially performed owing to suspicion of a hepatobiliary cause and demonstrated a small volume of free fluid within the pelvis (Figure 1), as well as echogenic stripe-like bands along the capsular margins of the liver and spleen (Figures 2 and 3). These features were highly suggestive of a pneumoperitoneum. On dynamic sonographic interrogation, the patient was negative for the “ultrasound Murphy’s sign” and denied focal tenderness in the right iliac fossa. There was also no sonographic evidence of gallstone pathology. An urgent chest radiograph subsequently confirmed the presence of a pneumoperitoneum projected beneath both hemidiaphragmatic contours (Figure 4).

The patient was urgently taken to theatre where laparotomy confirmed a perforated duodenal ulcer as the source of free air. The perforation was surgically managed with an excellent eventual outcome.

## Discussion

A hollow viscus perforation is an important diagnosis to detect an acute abdominal setting, owing to the high morbidity and mortality rates involved. The utility of ultrasound could potentially be useful as an adjunct to the diagnosis of a pneumoperitoneum. This is partly owing to user–operator dependability of the initial diagnosis as well as difficulty in recognizing typical sonographic features such as the peritoneal stripe sign, patient factors and differentiating between artifact caused by underlying organs and structures.

The use of CT is considered a “gold-standard” for demonstration of intraperitoneal free air.^1^ The concentration of extraluminal air bubbles in relation to the bowel wall, perivisceral fat stranding, presence of extraluminal fluid, thickening of the bowel wall and a focal defect in the bowel wall correlate closely with gastrointestinal tract perforations.^2^

Sonographic features of intraperitoneal free air rely on the detection of pockets of air in atypical locations within the abdominal cavity and the associated distortion of the ultrasound waves that it produces.^3^ The interface between the anterior abdominal wall and the adjacent peritoneal fluid results in a thin echogenic line on baseline ultrasound imaging.^3^ The abnormal presence of air in the abdomen scatters the ultrasound waves at the interface of soft tissue and air.^1^ This produces a high-amplitude linear echo known as the enhanced peritoneal stripe line which has been reliably established by various studies and a review article.^1,4–6^

The focal enhancement of the peritoneal stripe may be associated with posterior reflection artefacts.^3–5^ These reflection artefacts are a result of the appearance of air on ultrasound as an echogenic, reflective interface that obscures underlying anatomy.^6^ Long path reverberation artefacts are seen as horizontal stripes (Figure 3) created when the ultrasound waves reflect between air and the overlying fascial plane.^6^ Multiple air bubbles create echo tails which overlap and create a dirty shadow.^3,4^ This dark acoustic shadow arises when the different reflective surfaces created by air bubbles are less than half a pulse length apart and the returning impulses merge.^4,7^ The attenuation and diffuse reflection that result cause the returning ultrasound impulses to weaken and an acoustic shadow developes.^4,7^ A single row of bubbles produces echogenic lines that taper posteriorly and in turn gives rise to the appearance of comet tail artefacts. When these lines do not taper and instead maintain their width, these artefacts are known as “ring-down”.^4,6,8^

The sonographic findings of pneumoperitoneum were first identified in 1984.^9^ This was followed up by a 2007 study^10^ which determined that ultrasound is a more sensitive modality than plain radiography for detecting free air in the abdomen. The study found that plain radiography had a sensitivity of 79%, specificity of 64% and a positive predictive value of 96% for detecting pneumoperitoneum.^10^ Ultrasound imaging proved superior in terms of sensitivity at 93% and was at least comparable in terms of specificity at 64% and positive predictive value at 97%.^10^

A comparison study of 283 patients similarly noted the interference echo pattern and described the shifting phenomenon.^11^ As the echo transmission becomes interrupted by intraperitoneal air, the underlying structures fail to be delineated.^11^ In the lateral decubitus position where abdominal ultrasounds are frequently performed, the liver is obscured. In addition to echo interference, the presence of the shifting phenomenon^11^ is as equally important in determining the presence of intraperitoneal free air. As air can be shifted easily in the abdominal cavity, a change in the patient’s position results in a shift in the interference echo pattern.^11^ Using these two criteria, the study found that sonography diagnosed 9 out of 10 patients with documented acute perforated ulcer disease.^11^ Radiographic examination detected free air in eight of the documented cases.^11^

Optimal positioning for sonographic detection of intra-abdominal free air is generally considered to be with the patient being supine with the thorax slightly elevated at around 10–20 degrees.^7^ Some publications report that the optimal probe position should be within the right paramedian epigastric area in the longitudinal direction.^7^ In addition, it is suggested that the use of a linear-array transducer (10–12 MHz) would be superior to a standard curvilinear abdominal transducer (2–5 MHz) for detection of a pneumoperitoneum owing to its superior resolution in the near field where air accumulation occurs.^12^ However, this does not exclude the use of a standard curvilinear abdominal probe for the detection of intraperitoneal free air.

In addition to patient observational studies, there has been experimental studies to investigate the appearance of intraperitoneal free air within the abdomen. Cadaveric specimens and volunteers were subjected to graded intraperitoneal air injections to assess the characteristics of these air injections. Findings from these studies corroborate with previous sonographic demonstrations of intraperitoneal air as an enhanced echogenic line with posterior reverberation or ring-down artefact.^7,13^

Although the value of ultrasound in the detection of intraperitoneal free air has been reliably demonstrated, there are limitations to be considered. Hepato-diaphragmatic interposition of the colon, artefacts distal to an overlying rib and the adjacent lung^6,13^ can mimic the appearance of free air within the abdomen.^3^ There are, however, distinguishable features to separate the finding of intra-abdominal free air from a pseudo-pneumoperitoneum. For example intraluminal air is recognizable by its relationship with the adjacent bowel wall and moves with peristalsis.^6,14^

Sonographic artefacts from the adjacent parenchymal lung tissue and intervening ribs are identified by careful examination of the abdomen during inspiration and expiration. The shadows produced move with respiration and originate above the peritoneal line.^12,15^

This case outlines an example where the presence of pneumoperitoneum was accurately diagnosed with an ultrasound exam. The patient exhibited a bright enhancing peritoneal stripe with distinct ring-down artefacts (Figure 3). These findings corroborate with the current literature surrounding sonographic diagnosis of pneumoperitoneum.

## Conclusions

A pneumoperitoneum can have profound outcomes for patients and has the potential to be rapidly fatal. Multiple studies demonstrate that with minimal training, physicians and technologists with ultrasound experience can reliably recognize the signs of pneumoperitoneum on ultrasound with good accuracy. As such, this raises the possibility of the expanded use of ultrasound to detect free air within the abdomen in the acute abdominal setting.

## Learning points

1. Rapid diagnosis of a perforated hollow viscus is vital to ensure a good clinical outcome.
2. Bedside ultrasound could be a useful adjunct and has demonstrated reliability across a range of operators.
3. The presence of air in abnormal locations scatters the ultrasound waves at the interface of soft tissue and air resulting in an enhanced peritoneal stripe sign, ring-down artefacts and acoustic shadows resembling “comet tails”.
4. The shifting phenomenon of movement of free intraabdominal air with patient position changes is strong sonographic evidence to support the presence of free air in the abdomen.
5. A linear array transducer (10–12 MHz) would be preferable over the standard curvilinear probe (2–5 MHz) owing to its superior near-field resolution.

## Consent

Written informed consent for the case to be published (including images, case history and data) was obtained from the patient(s) for publication of this case report, including accompanying images.
